# Supplementary material for: Untargeted proteomics enables ultra-rapid variant prioritisation in mitochondrial and other rare diseases
Source: Genome Med. 2025 May 22;17:58. doi: 10.1186/s13073-025-01467-z (PMC12096736; doi:10.1186/s13073-025-01467-z)
Supplement: Supplementary file 2 — Additional file 2. Detailed clinical history of VC15 (UQCRC2) and VC24 (FARS2), Figure S1. Heatmaps and hierarchical clustering of the validation cohort (VC), Figure S2. Protein correlation between cell lines in the validation cohort (VC) and knockout cohort (KC), Figure S3. Mitochondrial abundance, citrate synthase abundance and activity, Figure S4. Relative Complex Abundance (RCA) of OXPHOS complexes in the validation cohort (VC), Figure S5. Orthogonal testing and analysis of undiagnosed cohort and supporting cohort samples. [file 13073_2025_1467_MOESM2_ESM.docx]

**Additional File 2**

**Clinical history**

VC15 – *UQCRC2*

VC15 was born at 36 weeks gestation by elective caesarean for placental insufficiency to consanguineous parents. VC15 had intra-uterine growth restriction, subtle dysmorphism, was hypoglycaemic and had persistent lactic acidosis despite maximum inotrope support. Cardiology review showed moderate pulmonary hypertension and pulmonary stenosis, and VC15 died on day 2. Respiratory chain enzyme testing showed an isolated Complex III deficiency in skeletal muscle and skin fibroblasts (**Additional file 3: Table S2**) plus a combined Complex III and I deficiency in liver. An Illumina HumanCytoSNP-12 array showed an absence of heterozygosity in 3.7% of the genome, including a 6.7Mb region on chromosome 16 and a 1.5Mb region on chromosome 20 containing mitochondrial Complex III subunit or assembly factor genes, *UQCRC2* and *UQCC1*, respectively. Candidate gene sequencing of *UQCRC2* and *UQCC1*, identified a homozygous likely pathogenic variant in *UQCRC2* (**Additional file 1: Table S1**).

VC24 – *FARS2*

VC24 presented with severe intra-uterine growth restriction and hyperlactataemia and died in the neonatal period. VC24 had an older sibling who died in similar circumstances, without DNA or tissue available. Exome sequencing and segregation testing identified compound heterozygous likely pathogenic variants in *FARS2* (**Additional file 1: Table S1**). Fibroblast respiratory chain enzyme testing and mito-cocktail western blotting demonstrated loss of Complexes IV and I (**Additional file 3: Table S2**), consistent with a *FARS2* disorder.

**Additional file 2: Figure S1**. **A –** Heatmap and hierarchical clustering of OXPHOS subunits by complex and assembly factors in the validation cohort (VC) using fold-change values calculated using t-test analysis from baseline imputed proteomic dataset. **B –** Topographical heatmap using fold-change values calculated from t-test analysis using baseline imputed proteomic dataset for isolated defects in the validation cohort (VC). PDB ID: 5LDW (CI), 1ZOY (CII), 1BGY (CIII), 5Z62 (CIV), 7AJD (CV).

**Additional file 2: Figure S2**. **A –** Pearson correlation between validation cohort (VC) and knockout cohort (KC) using fold-change values for OXPHOS subunits annotated with MitoCarta3.0 calculated from t-test analysis using baseline imputed proteomic dataset. **B –** Pairwise correlation of fibroblast line from validation cohort (VC) and equivalent HEK293T knockout cohort (KC) from baseline imputed proteomic dataset for mitochondrial proteins highlighting the expected affected complex.

**
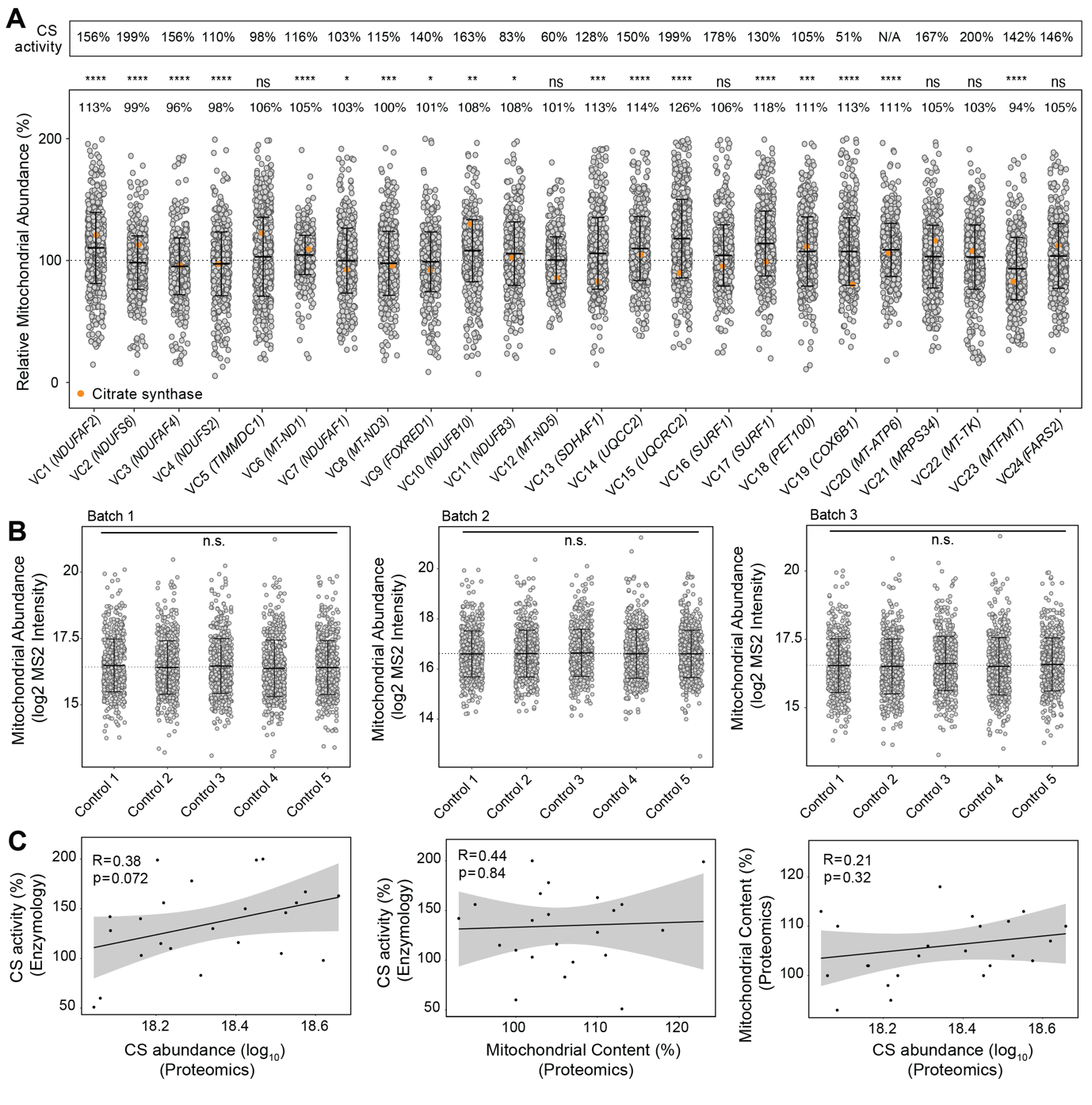
 Additional file 2: Figure S3**. **A –** Citrate synthase (CS) activity of fibroblast lines relative to controls and relative abundance of mitochondrial proteins (MitoCarta3.0) for the validation cohort (VC) compared to controls depicting variable abundance of mitochondrial levels across cell lines. Middle bar represents mean mitochondrial abundance. Upper and lower bars represent 95% confidence interval. Significance was calculated from a paired t-test between the mitochondrial means. Orange dot = citrate synthase protein abundance. **** = p<0.0001, *** = p<0.001, ** = p<0.01, * = p<0.05, ns = not significant, *p*>0.05. **B –** Relative abundance of mitochondrial proteins (MitoCarta3.0) in the five controls used in each batch of the validation cohort (VC) showing no significant changes using an ANOVA test. **C –** Pearson correlation between CS activity from enzymology and CS abundance from proteomics (left), CS activity from enzymology and mitochondrial content from proteomics (middle); and mitochondrial content from proteomics and CS abundance from proteomics (right).


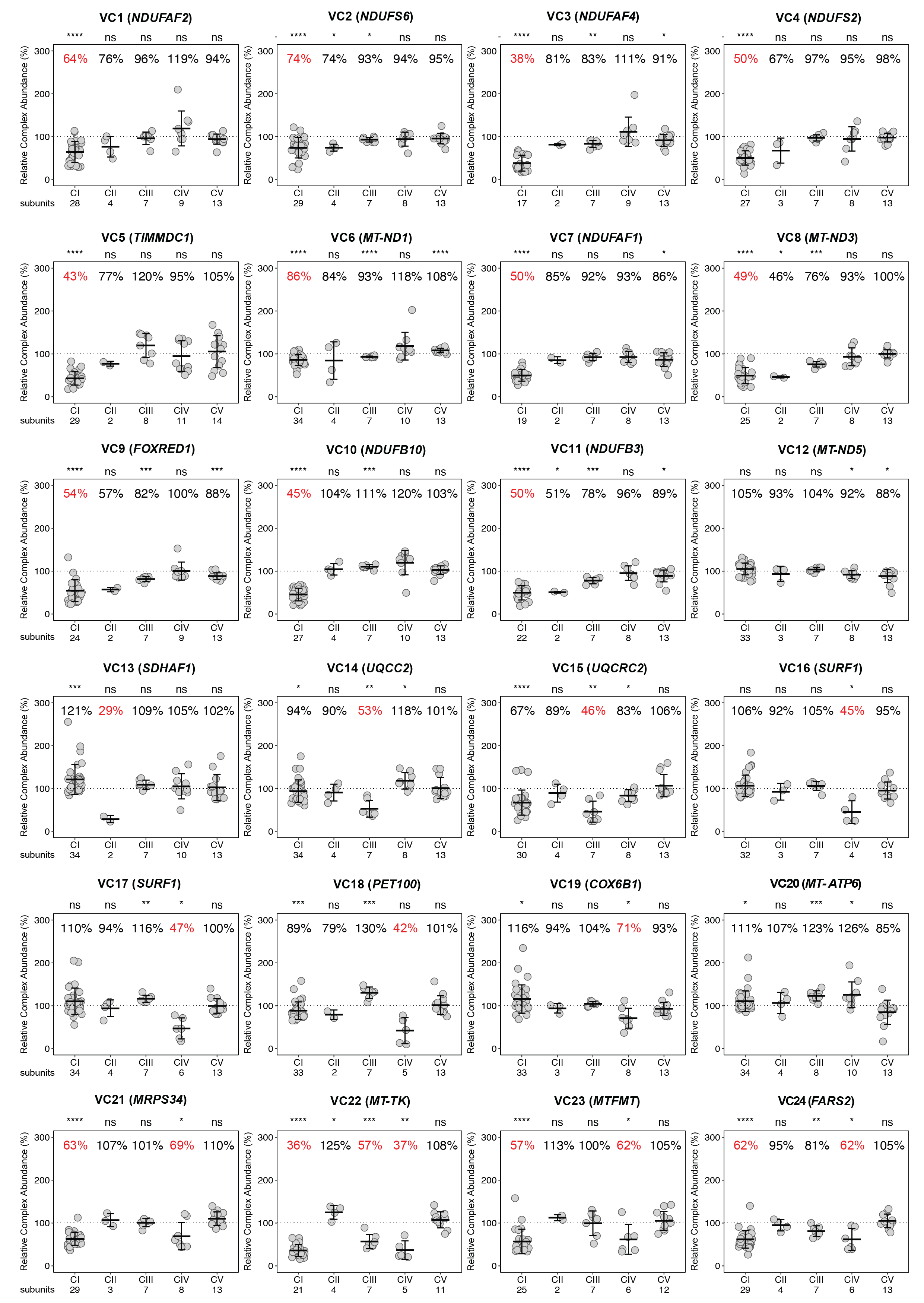
 **Additional file 2: Figure S4**. Relative Complex Abundance (RCA) of OXPHOS complexes in the validation cohort (VC) from non-imputed proteomic dataset. Middle bar represents mean complex abundance. Upper and lower bars represent 95% confidence interval. Significance was calculated from a paired t-test between the individual protein means. **** = p<0.0001, *** = p<0.001, ** = p<0.01, * = p<0.05, ns = not significant, *p*>0.05.

**Additional file 2: Figure S5**. **A –** Abundance of the ATAD3A mutant peptide (p.Met288) relative to the canonical peptide (p.Thr288). **B –** Transcriptomic analysis of skeletal muscle for UDP4 (*CCDC47*) relative to controls showing strong reduction of *CCDC47* transcripts. **C –** cDNA analysis of UDP4 (*CCDC47*) using RNA extracted from skeletal muscle. Gel electrophoresis of full-length PCR products designed to amplify the entire open reading frame (ORF) show the presence of both missense transcripts and a second shorter one, missing 121 bp from the 3’ end of exon 4 from a donor-splice site five nucleotides upstream of the missense variant. **D –** Relative abundance of NDUFA10, HIBCH and QRLS1 proteins from whole cell fibroblasts in UDP5 (*NDUFA10*) compared to controls. Significance was calculated from an unpaired t-test between the means. **** = p<0.0001, ** = p<0.01. **E –** Relative Complex Abundance (RCA) of OXPHOS complexes and mitoribosome large (mtLSU) and small (mtSSU) subunits in *MRPL50* peripheral blood mononuclear cells (PBMCs)(left) and fibroblasts (right). Middle bar represents mean complex abundance. Upper and lower bars represent 95% confidence interval. Significance was calculated from a paired t-test between the individual protein means. **** = p<0.0001, *** = p<0.001, ** = p<0.01, * = p<0.05, ns = not significant, *p*>0.05. **F –** Sanger sequencing of cloned *NDUFAF6* amplicons from proband fibroblasts showing three transcripts, the canonical transcript, one using an alternate +4bp 5’ splice site and a transcript containing a cryptic exon. **G –** Oxford Nanopore long-read sequencing of *NDUFS8* amplicons generated by PCR from blood sample using a forward primer in exon 1 and a reverse primer in exon 7. The patient predominantly expresses transcripts with in-frame exon 6 skipping (r.373_501del; p.Ala125_Glu167del). Some residual canonical exon 5-6-7 is observed in the patient. Heterozygote parental samples also show transcripts with exon 6 skipping and canonical exon 5-6-7 splicing. Exon 6 skipping is absent from disease controls. **H –** Oxford Nanopore long-read sequencing of *NDUFS8* amplicons generated from blood by PCR using forward primer in exon 6 and a reverse primer in exon 7. Of the transcripts which splice canonically from exon 5-6 in the patient sample, approximately half splice canonically from exon 6-7 and half retain intron 6 (r.501_502ins[501+1_502-1]; p.168Alafs*11). Intron 6 retention encodes a premature termination codon, however, these transcripts are not predicted to be degraded by nonsense mediated decay. Parental samples also exhibit increased intron 6 retention relative to disease controls.
